# Microwave-assisted synthesis of amphoteric fluorescence carbon quantum dots and their chromium adsorption from aqueous solution

**DOI:** 10.1038/s41598-023-37894-4

**Published:** 2023-07-12

**Authors:** Hebat-Allah S. Tohamy, Mohamed El-Sakhawy, Samir Kamel

**Affiliations:** Cellulose and Paper Department, National Research Centre, 33 El Bohouth Str., P.O. 12622, Dokki, Giza, Egypt

**Keywords:** Nanoscale materials, Environmental sciences, Environmental chemistry, Green chemistry, Materials chemistry, Organic chemistry

## Abstract

The chromium adsorption behavior from aqueous solution by the amphoteric Janus nitrogen-doped carbon quantum dots (AJ–N–CQDs) was investigated. The pseudo-first-order and the second-order adsorption kinetics models were employed to analyze the experimental data; the second-order adsorption kinetics model presented a better correlation to the experimental data, suggesting a chemisorptions process. The values obtained in the pseudo-first-order are still suitable for describing the Kinetics of Cr(VI) sorption. These values elucidate the surface processes involving chemisorption and physisorption in the adsorption of Cr(VI) by AJ–N–CQDs. The R^2^ of the Boyd model gave a better fit to the adsorption data of AJ–N–CQDs (i.e., external diffusion), which means the surface processes involving external Cr(VI) adsorption by AJ–N–CQDs. The higher value of α may be due to the greater surface area of the AJ–N–CQDs for the immediate adsorption of Cr(VI) from the aqueous solution. AJ–N–CQDs have fluorescence spectra before and after Cr(VI) adsorption, indicating they are promising for chemical sensor applications.

## Introduction

Accumulation of agricultural waste is harmful to the environment. As a result, recycling is in demand. The primary component of sugarcane bagasse is cellulose, which comprises β-1,4-glycosidic linkages of the D-glucose unit^1–4^. Utilizing agricultural waste has recently undergone a more significant effort. One was employing bagasse as a feedstock to produce carbon-based materials. It is the most dependable source for producing valuable products by eco-friendly techniques to remove metal ions from wastewater^1,5^.

Carbon quantum dots (CQDs) and graphene (G) are two valuable products that can be produced from agricultural wastes. CQDs are 10 nm diameters with colossal surface area ball nanomaterials, while graphene quantum dots (GQDs) are a mixture of G with CQDs. The most marked functional groups are O–H, –C = O, and C–O–C. Several methods, such as hydrothermal and solvothermal heating of organic molecules, laser ablation of graphite, and pyrolytic carbonization, have been used to create GQDs. However, studies have revealed that microwave heating is suitable for developing more rapid and reasonably priced CQD synthesis procedures^5^.

Amphoteric Janus nitrogen-doped CQDs (AJ–N–CQDs) containing –O– and –N– functional groups are amphoteric Janus materials (AJMs) due to the presence of hydrophobic carbon cores shielded by the layer of hydrophilic –O– and –N–^2,6,7^. Polymeric compounds with hydrophobic and hydrophilic moieties are called AJMs^7^.

Furthermore, N–CQDs are fluorescent and eco-friendly nanomaterials successfully used in wastewater treatment^1,5^. Despite its toxic character, Cr(VI) is widely used in various industries, and its recovery from the corresponding liquid effluents is a primary target before its discharge to natural waters. Therefore, monitoring the environmental content of Cr(VI) is essential for public health. Several materials have found applications to remove and/or recover Cr(VI) from wastes, such as activated carbon, natural zeolites, carbon nanotubes, etc.^8^. Among them, the adsorption onto AJ–N–CQDs could be competitive due to their high adsorption efficiency due to the presence of N and O functional groups and their fluorescence properties, which make them a suitable detector for Cr(VI)^1,5^. Several conventional approaches, including electrochemical technology, chromatography, etc., have been used to detect Cr(VI). Sample preparation is arduous, complicated, and time-consuming in most of these approaches. The fluorescence detection method is considered the more powerful than other conventional techniques because of its high sensitivity and low cost.

Consequently, the present work used microwave energy to prepare different carbon nanomaterials (GQDs and AJ–N–CQDs) from bagasse. The produced N–GQDs have no fluorescence properties due to the large amount of G. AJ–N–CQDs have fluorescence properties, making them promising adsorbents and sensors for metal ions such as Cr(VI). The Kinetics of the adsorption process at different times and the fluorescence of AJ–N–CQDs with/ without Cr(VI) were studied. The results are promising for chemical sensor applications.

## Materials and methods

### Materials

Sugarcane bagasse (SB) obtained from the paper industry, Quena company, Egypt, was grounded to a mesh size of 450 microns. Dimethyl formamide (DMF) and citric acid (CA) are of analytical grades and used without further purification. Potassium chromate (99%) was purchased from Sigma Aldrich as a model material for Cr(VI) ions.

Adsorption tests were carried out with potassium chromate (15 mg/L; Sigma Aldrich 99%) as a model material for Cr(VI) ions in wastewater.

### Methods

#### Preparation of quantum dots (QDs)

QDs were prepared using domestic and lab microwaves; a mixture of the SB (1 g), DMF (20 ml), and CA (0.1 g) was placed in a domestic microwave at 750 W for 3 min. The final brownish-black powder was washed with water, filtered, and dried under a vacuum to produce GQDs^1,5^.

The same mixture was placed in the lab microwave at 100 °C, 750 W, for 135 min. The final solution was left to cool down at room temperature, followed by centrifugation, filtration, and drying under vacuum, after which AJ–N–CQDs-based fluorescence nanoparticles were obtained.

### Characterization and analysis

#### Fourier-transform infrared spectroscopy (FT-IR)

Fourier-transform infrared spectra were collected employing Mattson 5000 spectrometer (Unicam, United Kingdom) using the KBr disk method.

#### Transmission electron microscope (TEM) analysis

TEM images were taken with a JEOL JEM-2100 electron microscopy at an acceleration voltage of 120 kV.

#### Scanning electron microscopy with energy-dispersive electron spectroscopy (EDX-SEM)

The elemental distribution of GQDs and AJ–N–CQDs was investigated using the non-destructive energy dispersive X-ray (EDX) unit attached to scanning electron microscopy (JSM 6360LV, JEOL/Noran). For surface morphology, imaging was recorded using an accelerating voltage of 10–15 kV.

#### X-ray diffraction(XRD)

The crystallinity was studied by X-ray powder diffraction. The diffraction patterns were measured by Bruker D-8 Advance X-ray diffractometer (Germany), applying a 40 kV voltage and a 40 mA current employing copper (Kα) radiation (1.5406 Å).

1 $$  {\text{Cr}}.{\text{I}}\left( \%  \right) = \frac{{A_{1} }}{{A_{t} }} \times 100 $$

2 $$ {\text{d spacing }}\left( {{\text{nm}}} \right){ } = \frac{\lambda }{2 sin\;\theta } $$

where A_1_ = area of the crystalline domain, At = area of the total domain^9^.

#### Nitrogen gas adsorption measurements (BET)

The BET was evaluated by Brunauer–Emmett–Teller (BET) technique using St 2 on NOVA touch 4LX, Quantachrome Instruments. The samples were pre-heated at a high temperature (120 °C). These samples were measured at liquid nitrogen after existence gassed at 200 °C under the flow of N_2_ for 3 h. The Barrett-Joyner-Halenda (BJH) method was used to measure the pore size.

#### Thermogravimetric analysis (TGA/DTG)

TGA analysis was performed on the prepared polymer powder employing Perkin Elmer thermogravimetric analyzer by heating the sample to 1000 °C of 10 °C/min under a nitrogen atmosphere. Thermal decomposition kinetics was explored for both N–CQDs and AJ–N–CQDs samples. The thermal analysis data were recorded to determine the thermal degradation's activation energy (Ea). Equations (3) and (4) were applied under the Coats-Redfern approach.

3 $$ log \left[ {\frac{{1 - \left( {1 - \propto } \right)^{1 - n} }}{{T^{2} \left( {1 - \propto } \right)^{{}} }}} \right] = {\text{log }}\frac{{{\text{AR}}}}{\beta E} - \frac{{\text{E}}}{{2.303{\text{ RT}}}}\;{\text{n}} \ne {1} $$

4 $$ log \left[ {\frac{{ - \log \left( {1 - {\upalpha }} \right)}}{{T^{{2}{}} }}} \right] = {\text{log }}\frac{{{\text{AR}}}}{\beta E}\left[ {1 - \frac{{2{\text{RT}}}}{{\text{E}}}} \right] - \frac{{\text{E}}}{{2.303{\text{ RT}}}}{\text{n }} = { 1} $$

Plotting the relationship between {log_10_ [1-(1-α)1-n] / T^2^ (1-n)} and 1/T using various suitable n values should show a straight-line correlation following Eq. (3). For n = 1, the relationship between log {–log(1-α)] / T^2^} and 1/T was plotted following Eq. (4). As a result, the least square method was used by selecting several n values (ranging from 0 to 3), calculating the correlation coefficient (r) for each value of n, and estimating the standard error (SE). The frequency factor A was determined from the intercept (log AR/ßE) of the Coats-Redfern equation by the most suitable value of n, while the activation energy (E) was calculated from the slope (E/2.303R). Equation (5) was used to estimate the other kinetic parameters, such as enthalpy (∆H), entropy (∆S), and free energy change (∆G)^10^.

5 $$ \Delta H = E - RT; \Delta G = \Delta H - T\Delta S\;and\;\Delta s = 2.303 \left( {log\frac{Ah}{{kT}}} \right)R $$

where (h) and (k) are Planck and Boltzmann constants, respectively.

#### Fluorescence spectroscopy

The Spectrofluorometer model Jasco FP – 6500, Japan, evaluated fluorescence spectra with a light source: a Xenon arc lamp 150 Watt.

### Chromium adsorption study

Comparative removal efficiency (R %) and adsorption capacity (qe) of AJ–N–CQDs were studied using 20 mg of absorbent in 20 ml of Cr(VI) aqueous solution at a concentration (15 mg/L), pH ≈ 5, and at different contact times (15, 30, 45, 60, 120, 240, and 360 min). Equations 6 and 7 were used to evaluate the AJ–N–CQDs sorption efficiencies.

6 $$ R \% = \frac{{\left( {C0 - Ct} \right)}}{C0} \times 100 $$

7 $$ qe = \frac{{\left( {C0 - Ct} \right)}}{m} \times V $$

where C_o_ and C_t_ are the Cr(VI) concentrations (mg/L) in the solution before and after adsorption, respectively. V is the volume of solution (L), m is the amount (g) of the sorbent employed in the adsorption experiment^10^.

Zero order, Pseudo-first-order, and Pseudo-second-order can be determined from the Eqs. (8), (9) and ((10).

8 $$ {\text{C}}_{{\text{e}}} = {\text{ C}}_{{\text{o}}} {-}{\text{ K}}_{{\text{t}}} $$

9 $$ {\text{ln }}\left[ {{\text{q}}_{{\text{e}}} - {\text{ q}}_{{\text{t}}} } \right] \, = {\text{ ln qe }}{-}{\text{ K}}_{{1}} {\text{t}} $$

10 $$ \frac{t}{qt} = \frac{1}{K2qe2} - \frac{t}{qt} $$

where q_e_ and q_t_ are the amounts of Cr (VI) adsorbed (mg/g) at equilibrium sorption capacity and time t, respectively. C_e_ is the final concentration of the AJ–N–CQDs with t (contact time). K_1_ (min^−1^) is the Pseudo-first-order rate constant of adsorption. K_2_ is the rate constant of Pseudo second-order adsorption. Values of q_e2_ and K_2_ were calculated from the slope and intercept plot of t/qt against t, respectively^6^.

The Weber–Morris intra-particle diffusion can be determined from Eq. (11).

11 $$ {\text{qt }} = {\text{ K}}_{{3}} {\text{t}}^{{{1}/{2}}} + {\text{ C}} $$

where K_3_ is the intra-particle diffusion rate constant and C is the slope which represents the thickness of the boundary layer^5^.

The Boyd, and Elovich models can be determined from Eqs. (12) and (13), respectively^11^.

12 $$ {\text{B}}_{{\text{t}}} = \, {-}0.{4977 }{-}{\text{ ln}}\left( {{1}{-}{\text{F}}} \right) $$

13 $$ qt = \frac{\ln \alpha b}{b} - \frac{1}{b}\ln t $$

where B_t_ is a mathematical function of F, equivalent to qt/qe, representing the fraction of adsorbate adsorbed at different times. Also, α is the initial rate of adsorption (mg/g/min), and b is related to the extent of surface coverage and activation energy for chemisorption (g/mg). A plot of qt against ln t yields a straight line with α and b determined using the slope (1/b) and intercept (ln αb/b), respectively^12^. The Elovich kinetic model can study the adsorption rate based on absorption capacity on heterogeneous surfaces^13^. This model is used further to describe the pseudo-second-order kinetic (i.e., chemisorptions), assuming that the sorbent surface is energetically heterogeneous^14^.

## Results and discussion

Fluorescent carbon quantum dots (CDs) are synthesized and employed as adsorbent and fluorescent chemosensors for the selective detection of Cr(VI) ions. FT-IR, TEM, SEM/EDX, XRD, and TGA/DTG investigated the structure, size, and morphology.

### Structure characterization

#### FTIR spectroscopy

**Figure1:**
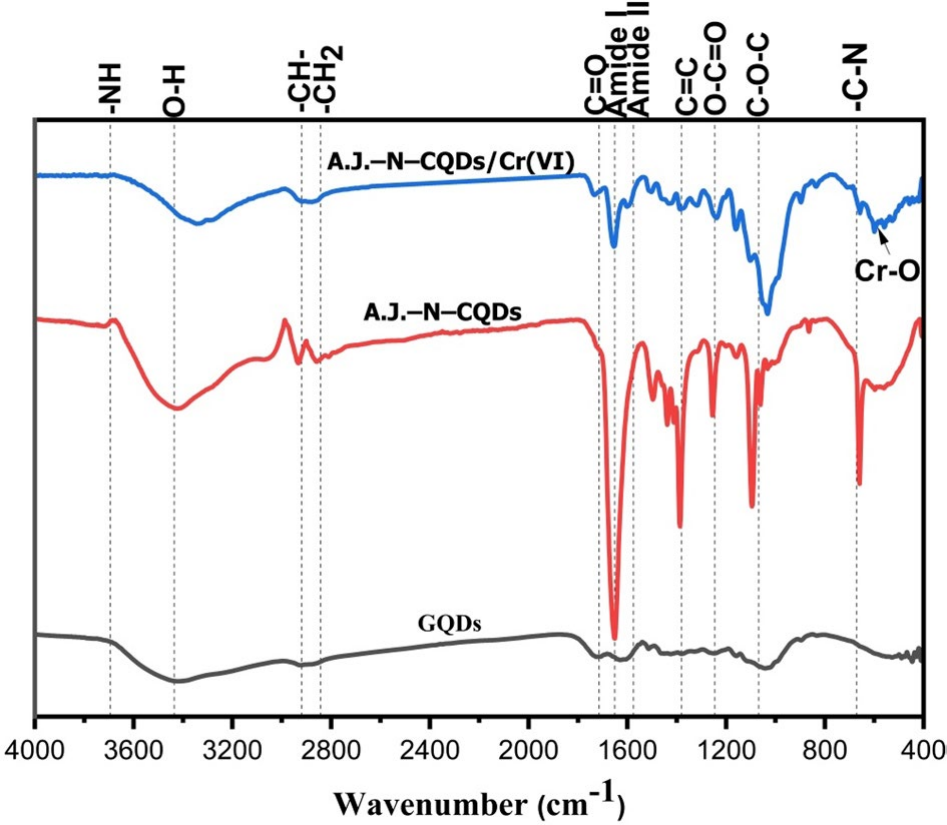
FTIR spectra of GQDs, AJ–N–CQDs, and AJ–N–CQDs/Cr(VI).

The FTIR spectra of the prepared GQDs, as shown in Fig. 1, revealed a broad peak at absorption band 3426.06 cm^-1^ attributed to the vibration contributions of O–H groups. The AJ–N–CQDs and AJ–N–CQDs/Cr(VI) showed separated peaks for O–H and –N–H at 3721.96, 3422.61 and 3344.41, 3286.30 cm^-1^, respectively. For GQDs, AJ–N–CQDs, and AJ–N–CQDs/Cr(VI), the peaks between 2919.87–2935.25, 2858.78–2878.5, 1654.70–1743.43, 1496.47–1656.65, 1438.25–1596.86, 1381.80–1387.08, 1238.15–1254.67, and 1033.72–1094.95 cm^-1^ were attributed to–CH–, –CH_2_, C = O, amide I, amide II bands, C = C, O–C = O, and C–O–C, respectively. There is an additional C–N group for AJ–N–CQDs and AJ–N–CQDs/Cr(VI) between 659.71 and 661.49 cm^-1^. The presence of amine groups (N–H and C–N) for AJ–N–CQDs and AJ–N–CQDs/Cr(VI) confirmed the formation of aminated CQDs^1,2,5^. At the same time, the O–H of N–GQDs at 3426.06 cm^−1^ was shifted to 3422.61 cm^−1^ for AJ–N–CQDs. This shift is attributed to the stronger intermolecular H-bonding. The calculated mean hydrogen bond strengths (MHBS) were 0.90 and 0.98 for N–GQDs and AJ–N–CQDs, respectively, confirming the formation of this intermolecular H-bonding^10^.

The relative absorbance (RA) of the characteristic –N–H are 1.09 and 0.95, and for C–N group are 3.62 and 0.97 for AJ–N–CQDs and AJ–N–CQDs/Cr(VI), respectively, confirmed the high content of nitrogenized groups in the prepared AJ–N–CQDs than AJ–N–CQDs/Cr(VI), which due to the bonding of Cr(VI) with the aminated groups. The Cr(VI) reduction in the presence of amide I and amide II in AJ–N–CQDs is apparent for AJ–N–CQDs/Cr(VI)) by a decreased intensity of the C = O peak (1743.43 cm^-1^) than AJ–N–CQDs (1654.70 cm^-1^(. The RA of the O–H group was 0.87 and 0.96 for AJ–N–CQDs and AJ–N–CQDs/Cr(VI), respectively. The fluorescence inner filter effect further supports this in the fluorescence spectra. The FTIR for AJ–N–CQDs/Cr(VI) showed an additional peak at 599.78 cm^-1^ assigned to Cr–O bonds due to the adsorbed Cr(VI).

#### Morphological analysis and elemental composition

**Figure 2:**
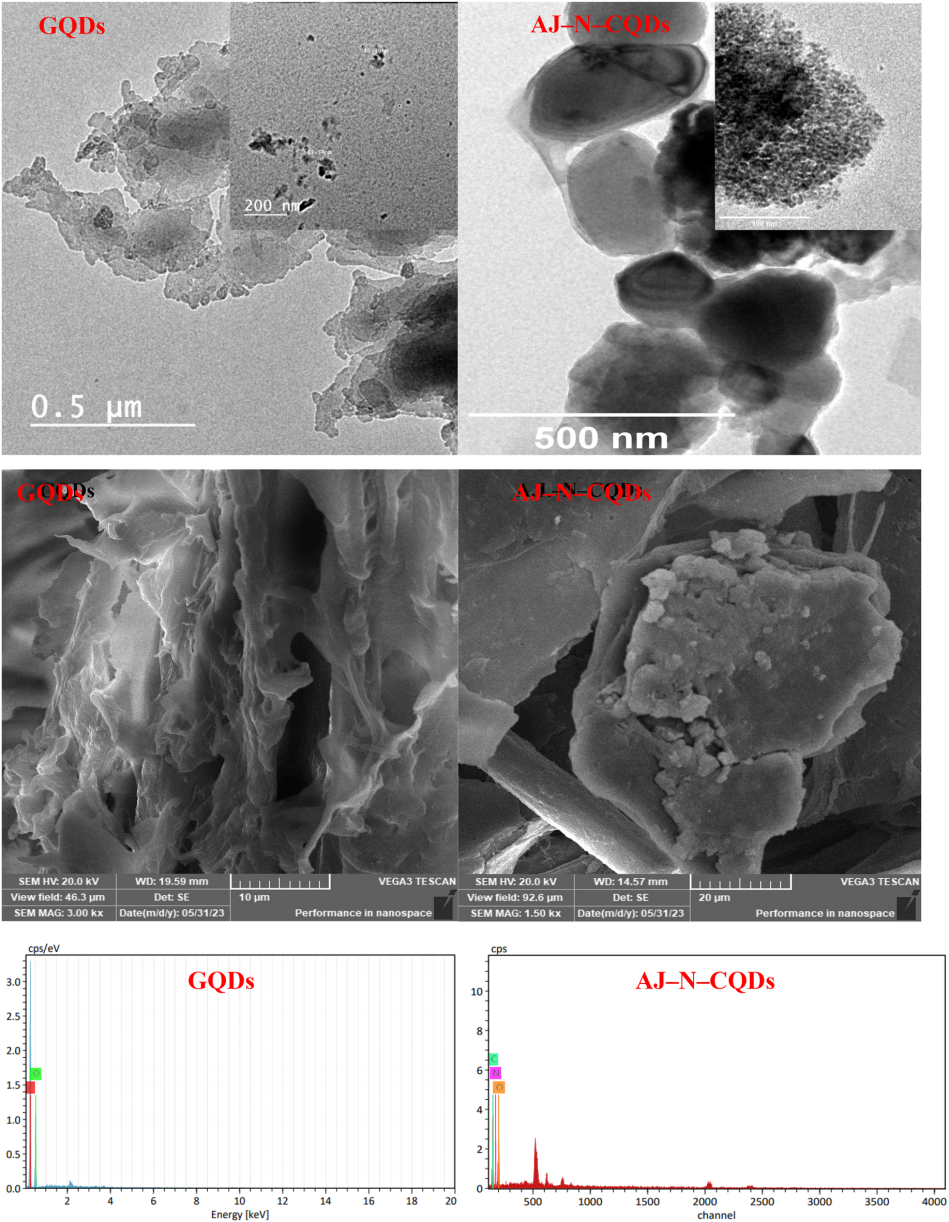
TEM, SEM images and EDX analysis of GQDs and AJ–N–CQDs.

From TEM analysis (Fig. 2), graphene (G) sheets were observed for GQDs with small amounts of dots, while AJ–N–CQDs depicted pure nanoparticles with spherical regularity without graphene sheets. The average diameters of nanoparticles were 13.34 and 8.42 nm for GQDs and AJ–N–CQDs, respectively. In addition, the particle size distribution is wide and narrow for GQDs (8–15 nm) and AJ–N–CQDs (between 8 and 9 nm), respectively. Using a domestic microwave, TEM images revealed the presence of GQDs^5^. While by using a lab microwave with changing the conditions produced pure CQDs. Figure 2 shows that the number of dots in AJ–N–CQDs is higher than in GQDs, confirming the lab's efficiency over the domestic microwave for preparing pure AJ–N–CQDs.

SEM images showed fluffy sheets of GO for GQDs. The dots didn’t appear here due to their low number. In addition, AJ–N–CQDs showed crumpled precipitated structures, which didn’t show any dots due to the small particle size of the AJ–N–CQDs and the agglomeration existing because of the storage of the sample. EDX results showed that the AJ–N–CQDs are nitrogenized with nitrogen content of ~ 2.90% while, GQDs are non-nitrogenized (Fig. 2).

#### XRD analysis

**Figure 3:**
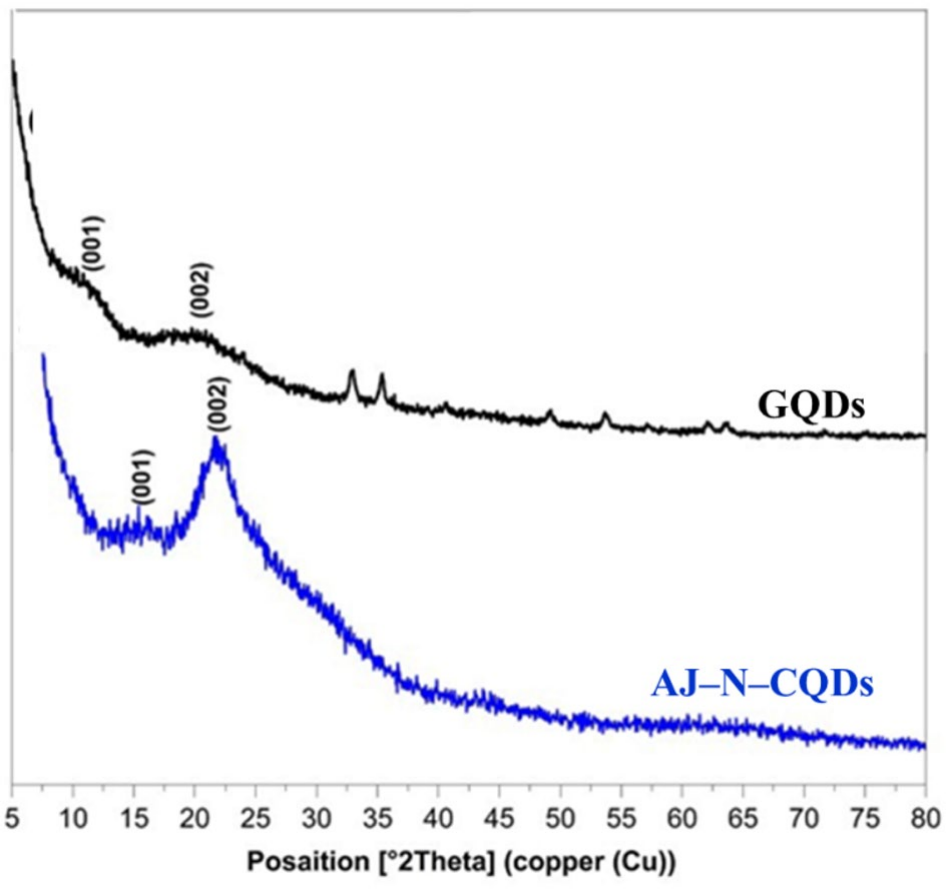
XRD pattern of GQDs and AJ–N–CQDs.

The XRD pattern of GQDs, as shown in Fig. 3, revealed peaks at 10.84 and 22.80° related to the (001) and (002) planes, respectively, due to the presence of GO with the d $\approx$ 0.18 nm^5^. The AJ–N–CQDs showed a broad peak at 22.02 related to the (002) plane (JCPDS card no. 26–1076)^15^. The d value of AJ–N–CQDs (0.20 nm) is higher than GQDs (0.18 nm). This may be due to the presence of N–and O–groups, which enlarge the spacing of AJ–N–CQDs^16^. The calculated Cr.I. % of GQDs and AJ–N–CQDs were 72.04 and 94.68%, respectively.

#### BET surface area determination

**Figure 4:**
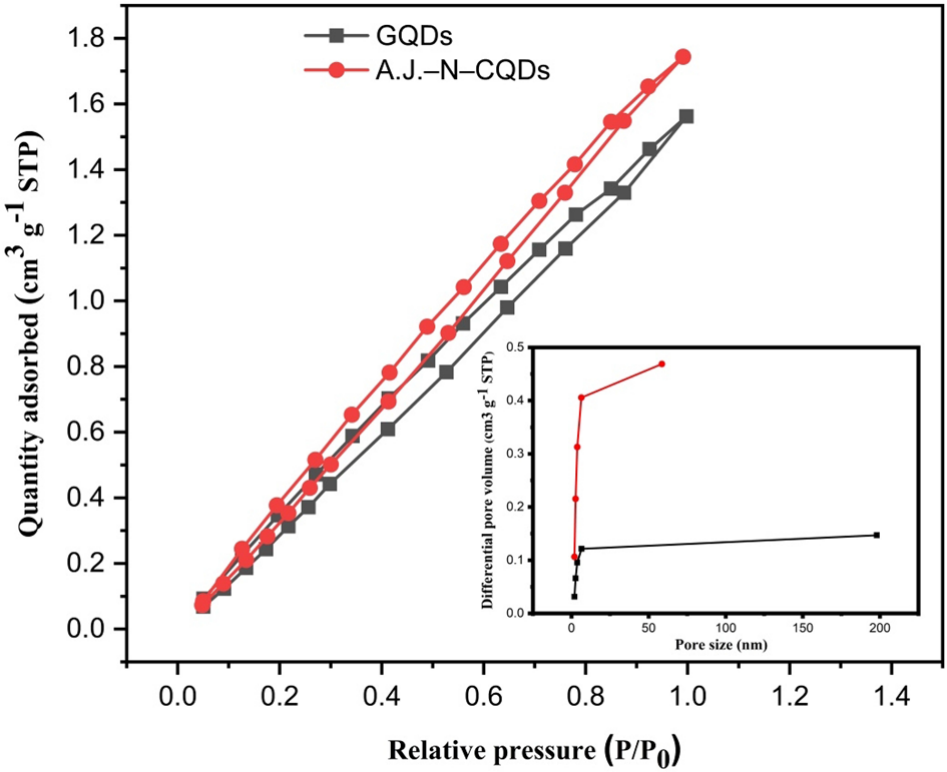
Nitrogen adsorption/desorption isotherms (the inset shows the pore size distribution).

The nitrogen (N_2_) adsorption isotherm was analyzed using the Brunauer- Emmett- Teller (BET) method to calculate the specific area. Figure 4 illustrates the N_2_ adsorption isotherms and pore size distribution. The pore size and distribution provide the necessary information about the chemical and physical interaction of the adsorbed metal with the adsorbent surface. The N_2_ adsorption isotherm was used to measure the BET surface and volume of the pores.

The surface area of GQDs and AJ–N–CQDs are 208.48 and 675.75 m^2^/g, while the average pore radii are 1.63 X 104 and 1.60 X 107 nm, respectively. The results showed that the surface area of AJ–N–CQDs is higher than that of GQDs. In general, a high surface area can provide sufficient adsorption sites. At the same time, increasing the number of pores and volume of the sample adequately makes it contact with the Cr(VI), which can promote the improvement of the adsorption performance. This may be one reason for the difference in fluorescence properties.

#### Thermal study

**Figure 5:**
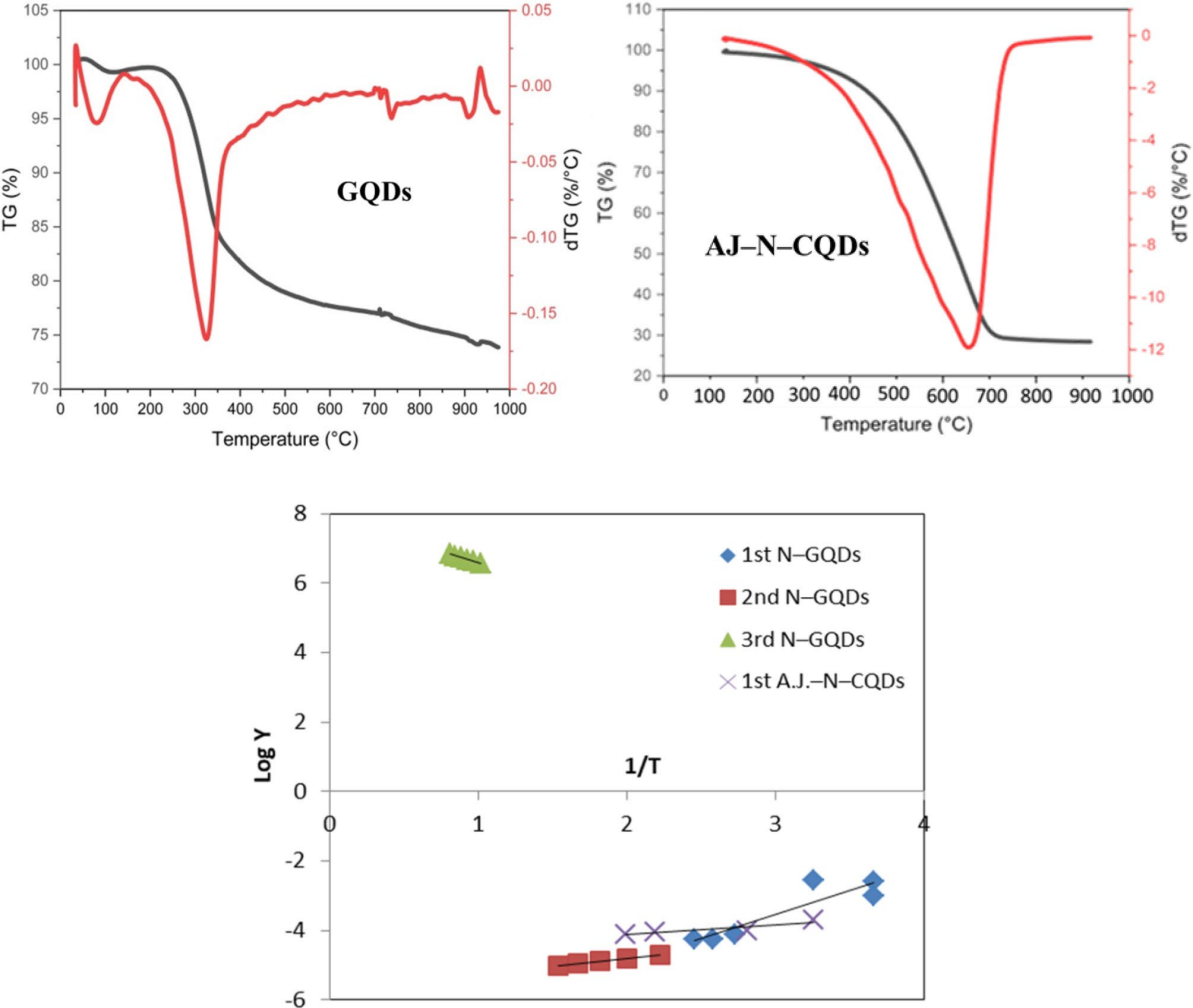
TGA/DTG of GQDs and AJ–N–CQDs, and their kinetics.

The TGA/DTG of GQDs, as shown in Fig. 5, revealed three decomposition steps, while AJ–N–CQDs revealed one decomposition step with a mass loss (ML) of 72.92 and 69.79%, respectively, at 1000 °C, due to the non-volatile contents. The first ML for GQDs was 34.46–143.38 °C with a maximum temperature of 76.79 °C and an average ML of 3.14% due to the moisture content. The second ML stage is between 176.80 and 394.98 °C, with a maximum temperature of 323.99 °C and an average ML of 13.68%, due to the pyrolytic degradation. Finally, the third ML stage is between 709.13 and 1000 °C, with a maximum temperature of 734.55 °C and an average ML of 26.10%, due to the decomposition of the residual carbon^9,16^.

At the same time, the first ML of AJ–N–CQDs was in the range of 68.38–189.32 °C with a maximum temperature of 163.90 °C and an average ML of 61.79% due to the moisture content. As it is seen, the AJ–N–CQDs need more temperature above 1000 °C to complete their degradation due to their high thermal stability^6^.

According to Table 1, all samples under investigation have values for ΔH for the GQDs decrease from about 9.40 kJ mol^-1^ at the first stage of the reaction to about 3.94 kJ mol^-1^ at the second stage, indicating a shallow energy requirement as the reaction progresses (Ea decreased from 12.31 to 8.90 kJ mol^-1^). With lowering n (from 1.5 to 0.5) values, the ΔH was reduced (3.94 kJ mol^-1^). After that, with an increasing n value (2.5), the ΔH was increased (16.89 kJ mol^-1^), indicating a high energy requirement for the final stage (Ea $\approx$ 25.26 kJ mol^-1^)^1,6^. According to the ΔG observations, the AJ–N–CQDs are more non-spontaneous and require external heat input than the GQDs, which improves their high thermal stability^4^.

**Table 1:** TGA/DTG data of N–GQDs and AJ–N–CQDs.

Sample	Stage	n	R^2^	A	$\Delta \mathrm{H}$	$\Delta \mathrm{s}$	$\Delta \mathrm{G}$	SE	E_a_
N–GQDs	1st	1.5	0.945	0.35	9.40	− 0.25	098.59	25X10^–2^	12.31
	2nd	0.5	0.997	2.84	3.94	− 0.24	148.43	79X10^–3^	08.90
	3rd	2.5	0.999	0.26	16.89	− 0.26	285.04	28X10^–3^	25.26
AJ–N–CQDs	1st	0.5	0.998	2.44	2.70	− 0.24	107.86	12X10^–2^	06.34

#### Fluorescence spectra

**Figure 6:**
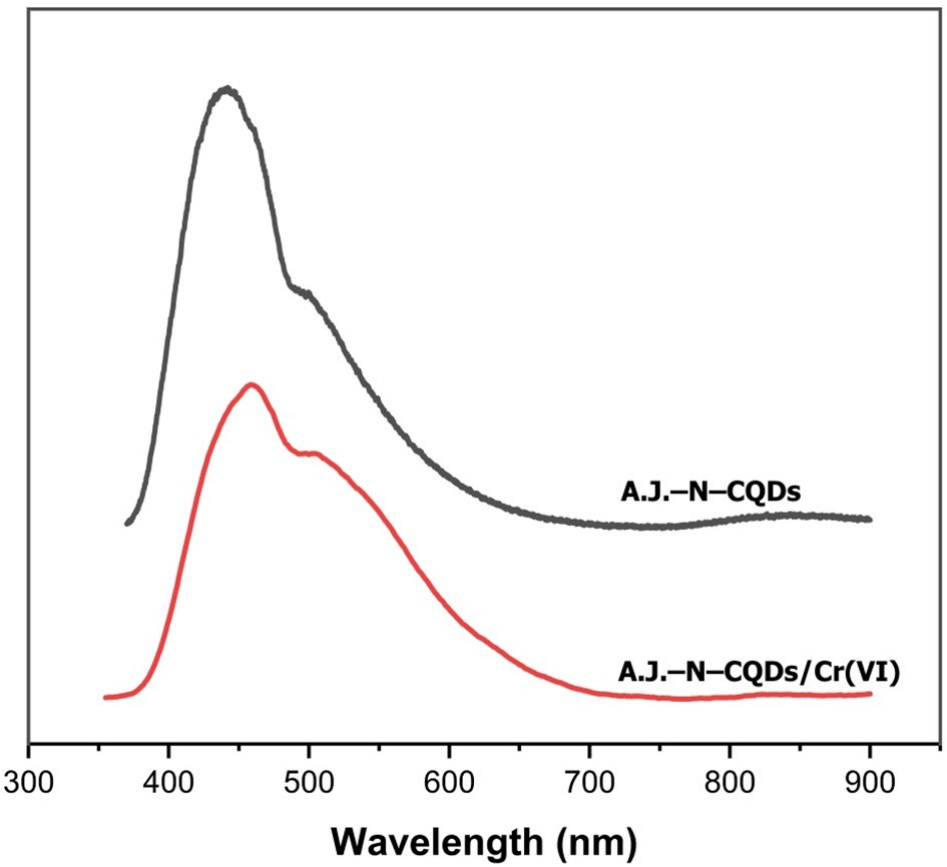
Fluorescent spectra of AJ–N–CQDs and AJ–N–CQDs/Cr(VI).

Firstly, GQDs did not produce any fluorescence spectra. This may be due to the presence of a large number of graphene, which blocks the fluorescent dots in the GQDs. So, in this study, we will use AJ–N–CQDs for Cr(VI) adsorption. Figure 6 shows the fluorescence emission spectra of the AJ–N–CQDs and AJ–N–CQDs/Cr(VI). They were excited at 350 nm and showed a maximum emission wavelength of 442.0 and 459.0 nm, respectively. This blue shift emission could theoretically prove using of these materials as sensors. Fluorescence emission is caused by C = O/C = N moieties of the CQDs' surfaces. The emission peaks at 511.0 and 499.5 nm are derived from the oxygen vacancy for AJ–N–CQDs and AJ–N–CQDs/Cr(VI), respectively. The oxygen vacancy defects in the AJ–N–CQDs/Cr(VI) case are almost hidden and may be due to Cr(VI) adsorption. The high RA of OH groups prove this oxygen vacancy from FTIR. The fluorescence quenching efficiency (FQE) was determined using the following formula^17^:

$$ {\text{FQE }} = \frac{{F0{-}F}}{F0} $$

where F_0_ and F refer to the F.I. of AJ–N–CQDs and AJ–N–CQDs/Cr(VI) Cr(VI), respectively. The calculated FQE was 49.57%, indicating relatively high sensitivity. The interaction between nitrogenized and oxygenated surface functionalities (–COOH, –OH, and –NH_2_) of AJ–N–CQDs/Cr(VI) was found to be responsible for the FQE^18^. The reduction in fluorescence intensity of AJ–N–CQDs/Cr(VI) compared to AJ–N–CQDs is due to IFE. The Cr(VI) ions absorb light radiation in the same range of wavelength of AJ–N–CQDs. As a result, the intensity is reduced, reducing the fluorescence intensity of AJ–N–CQDs/Cr(VI)^19^. In addition, because of high FQE, these findings validated the efficiency of AJ–N–CQDs synthesized from bagasse as an excellent material for further utilization in chemical sensing applications.

#### Adsorption study

**Figure 7:**
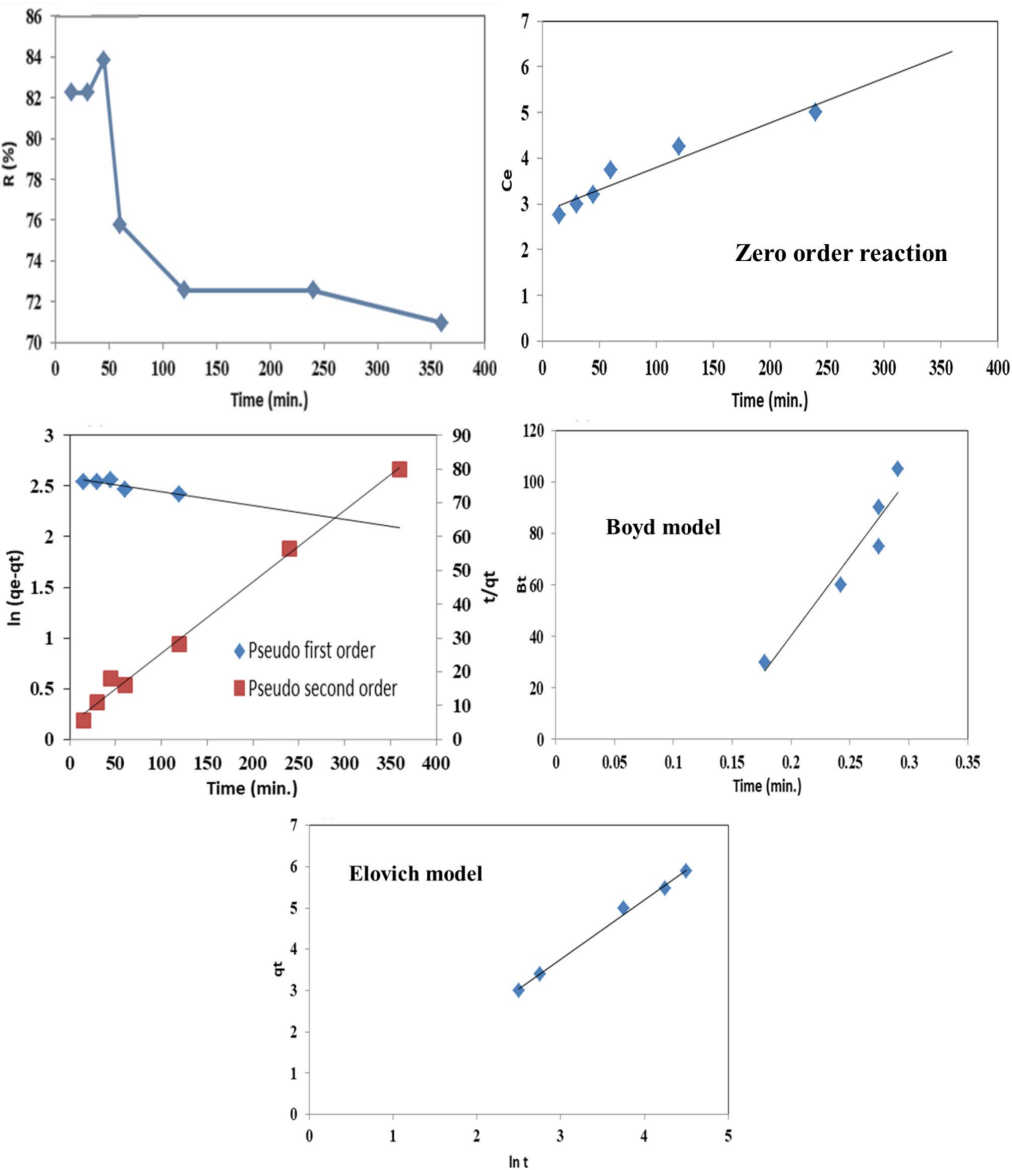
Effect of contact time, Zero order reaction, pseudo-first-order & the pseudo-second-ordere, Boyd model, and Elovich model for Cr(VI) adsorption by AJ–N–CQDs.

In this study, we choose AJ–N–CQDs with fluorescent properties to remove Cr(VI). The effect of contact time on the adsorption efficiency of AJ–N–CQDs was studied at different times, namely 15, 30, 45, 60, 120, 240, and 360 min. As shown in Fig. 7, the affinity of AJ–N–CQDs towards Cr(VI) was not the same, and the removal of Cr(VI) by AJ–N–CQDs was found to be fast at first due to the presence of more free functional groups then became slow. There was no remarkable increase in the adsorption rate observed after 45 min. So, the optimum time for Cr(VI) adsorption on AJ–N–CQDs at 25 °C is 45 min. Cr(VI) adsorption to AJ–N–CQDs was 83.85% and stable until 45 min. The adsorption may be due to high nitrogen and oxygen content. After 45 min, the adsorption rate decreased due to the leaching process^1,6^.

The Zero order reaction did not make it suitable for describing the Kinetics of Cr (VI) sorption on AJ–N–CQDs which assumes that increasing the concentration of reactants does not affect the magnitude of the reaction rate^20^. Therefore, pseudo-first-order and pseudo-second-order equations are utilized to model the Cr (VI) Kinetics on AJ–N–CQDs. Concerning the values of R^2^ presented in Table 2, it is seen that the pseudo-second-order model gave a better fit to the adsorption data of AJ–N–CQDs (chemical bonds in the adsorption). However, the values obtained in the pseudo-first-order are still suitable for describing the Kinetics of Cr (VI) sorption. These values elucidate the surface processes involving chemisorption and physisorption in the adsorption of Cr (VI) by AJ–N–CQDs^5^.

**Table 2:** Comparison between the estimated adsorption rate constants, rate constants, and correlation coefficients associated with the pseudo-first-order, the pseudo-second-order, Boyd model, and Elovich model for Cr(VI) adsorption by AJ–N–CQDs.

Kinetic model	Parameter	AJ–N–CQDs
Zero order reaction	K_0_ R^2^	53X10^–3^ 0.7459
Pseudo-first order	q_exp._ (mg/g) q_Calc._ (mg/g) K_1_ R^2^	12.57 13.44 19X10^–3^ 0.7729
Pseudo second order	q_Calc._ (mg/g) K_2_ R^2^	1.29 49X10^–2^ 0.9941
Boyd model	Slope Intercept R^2^	15X10^–3^ 13X10^–2^ 0.9190
Elovich model	a (mg/g/min.) b (g/mg) R^2^	7.14X10^–6^ 16X10^–2^ 0.8383

The Boyd model shows that the linear adjustment does not cross the axis at the origin, indicating that external diffusion was not only the rate-controlling step. Therefore, the lowest slope for the Boyd model is relatively associated with a strong effect of intra-particle diffusion. At the same time, R^2^ of the Boyd model gave a better fit to the adsorption data of AJ–N–CQDs (external diffusion). These values elucidate the surface processes involving external and intra-particle diffusion in Cr(VI) adsorption by AJ–N–CQDs. The higher value of α may be due to the greater surface area of the AJ–N–CQDs for the immediate Cr(VI) adsorption from an aqueous solution^5,21^.

#### Comparative study

In our study we used a Cr(VI) of 15 mg/L to stimulate the pollutant water, so at 100% removal of Cr(VI) the adsorbent capacity will be 15 mg/g. The maximum Cr(VI) removal % of AJ–N–CQDs is 83.85% by AJ–N–CQDs which is higher than the adsorption of other adsorbents stated in the literature (Table 3). For example, R % was 48, 45, and 37% for carbon dots modified mesoporous organosilica as an adsorbent for the removal of Hg(II), Cu(II), and Pb(II), respectively^22^. In addition, the maximum R % of Cr(VI) by TiO_2_/CQDs was 43.39%^23^.

**Table 3:** The maximum adsorption capacity (Q, mg/g) of several heavy metal ions on CQDs.

Adsorbent	Metal ion	Q	Removal %	Conditions	Reference
CQDs linked with mesoporous organ silicate	Hg(II) Cu(II) Pb(II)	56 53 43	48 45 37	Initial concentration 10^−3^ M, pH ≈ 4, and Temperature 25 °C	^22^
TiO_2_/CQDs	Cr (VI)	55	43.39	5 mg/l, pH ≈ 2, and Temperature 25 °C	^23^
A.J.–N–CQDs	Cr (VI)	12.57	83.85	15 mg/l, pH 5, and Temperature 25 °C	Our study

## Conclusions

This research reported a simple microwave method for preparing nitrogen-doped CQDs (AJ–N–CQDs) from bagasse, citric acid, and DMF. The AJ–N–CQDs have a high adsorption capacity and high sensing to Cr(VI). It is shown that Cr(VI) adsorption on AJ–N–CQDs is rapid in the first 45 min due to the presence of high nitrogen and oxygen functional group. FTIR-Spectra, XRD, TEM, and SEM/EDX confirmed the formation of AJ–N–CQDs. The morphology and BET studies confirmed that AJ–N–CQDs had a porous structure, significantly affecting their absorption properties. The kinetic absorption studies of as-prepared AJ–N–CQDs for Cr(VI) followed the Pseudo-second-order model. The fluorescence intensity of AJ–N–CQDs was reduced with Cr(VI) adsorption. Therefore, the present work offers a practical strategy toward a simple production method for preparing AJ–N–CQDs and their potential applications for Cr(VI) adsorption and chemosensor of Cr(VI).

## Data Availability

Data are available on request from the corresponding author.
